# Editorial: Viral diseases in swine

**DOI:** 10.3389/fcimb.2025.1599457

**Published:** 2025-05-01

**Authors:** Ke Liu, Muddassar Hameed, Zhiyong Ma

**Affiliations:** ^1^ Shanghai Veterinary Research Institute, Chinese Academy of Agricultural Science, Shanghai, China; ^2^ Fralin Life Science Institute, Virginia Tech, Blacksburg, VA, United States

**Keywords:** swine diseases, vaccine, drug, diagnostics, antimicrobial resistance, mechanisms

Swine diseases, especially swine viral diseases, remain a persistent threat to global food security and the swine industry, causing significant economic losses and compromising animal welfare. The emergence of novel pathogens, such as African swine fever virus (ASFV), and the evolution of existing viruses like porcine reproductive and respiratory syndrome virus (PRRSV) and porcine epidemic diarrhea virus (PEDV), underscore the urgent need for innovative approaches to detection, prevention, and treatment. This Research Topic brings together cutting-edge studies that address critical gaps in our understanding of swine viral diseases, offering new insights into pathogenesis, diagnostics, and therapeutic strategies.

## Breakthroughs in rapid and sensitive diagnostics

Advances in diagnostic tools are pivotal for early disease control. Cao et al. present a CRISPR/Cas12a-based assay integrated with enzymatic recombinase amplification (ERA) for ASFV detection, achieving a sensitivity of 10 copies per reaction. This method offers a portable, field-deployable solution, crucial for resource-limited settings. Song et al. further demonstrate the utility of recombinase-aided amplification (RAA) combined with lateral flow dipsticks for Senecavirus A, enabling visual detection within 17 minutes. These studies exemplify the potential of isothermal amplification technologies to revolutionize point-of-care diagnostics.

## Innovations in vaccine development

Vaccines remain the cornerstone of swine disease prevention. Chen et al. review the promise of multi-epitope vaccines, leveraging immunoinformatics to design constructs targeting conserved regions of variable pathogens like ASFV and PRRSV. Xie et al. extend this concept by evaluating recombinant adenovirus vaccines expressing GP3 and GP5 proteins of NADC34-like PRRSV, demonstrating robust humoral and cellular immune responses in pigs. These findings highlight the importance of subunit vaccines and adjuvant strategies in combating antigenically diverse viruses.

## Therapeutic strategies and drug development

The identification of novel antiviral compounds is equally critical. Li et al. explore chebulinic acid’s dual inhibitory effects on PEDV entry and viral main protease, offering a natural product-based therapeutic candidate. This work underscores the potential of phytochemicals in combating coronaviruses, aligning with global efforts to discover sustainable antiviral agents.

## Unveiling mechanisms of pathogens spread and resistance

A highlight of this Research Topic is the study by Jiang et al., which investigates the horizontal transmission of tigecycline resistance via the ISVsa3-ORF2-abh-tet(X4) circular intermediate in *Escherichia coli* from duck farms. This work highlights the role of mobile genetic elements in disseminating antibiotic resistance, emphasizing the need for vigilant monitoring to mitigate public health risks. Similarly, the analysis of virulence plasmids in edema disease-causing *E. coli* by Nemati et al. uncovers plasmid-mediated adhesion and toxin production, providing a foundation for targeted interventions against this economically devastating condition.

## Looking forward: bridging gaps and fostering collaboration

-While these studies advance our knowledge, several challenges persist. The rapid evolution of viruses necessitates continuous surveillance and adaptive strategies. Future research should prioritize:

1. Multi-omic Approaches: Integrating genomic, transcriptomic, and proteomic data to unravel host-pathogen interactions and immune evasion mechanisms.
2. Field-Validated Solutions: Translating laboratory breakthroughs into scalable diagnostic tools and vaccines for resource-limited settings.
3. One Health Framework: Addressing zoonotic risks and antimicrobial resistance through collaborative efforts across veterinary and public health sectors.
4. CRISPR Technology: Enable swine disease research and detection to enter a deeper level of molecular and cell biology.
5. Microfluidics-LAMP: Make the clinical detection of swine diseases faster, more accurate and more convenient.

This Research Topic serves as a testament to the interdisciplinary nature of swine disease research. By bringing together insights from virology, immunology, and epidemiology, it paves the way for a more resilient swine industry. We extend our gratitude to all authors and reviewers for their contributions, which collectively strengthen our capacity to tackle these pressing challenges.

